# Alteration of MR-DWI/ADC before and 24h after induction of chemotherapy in patients with lung cancer

**DOI:** 10.1186/1470-7330-14-S1-P34

**Published:** 2014-10-09

**Authors:** O Sedlaczek, C Wiedemann, C Gruellich, U Klingmüller, HU Kauczor, HP Schlemmer

**Affiliations:** 1 Department of Radiology, German Cancer Research Center, Heidelberg, Germany

## Background

Diffusion weighted MRI (MR-DWI) is frequently used in oncologic therapy monitoring as it is known to predict therapy response much earlier, than measurements of tumour size alone. In several studies the outcome of chemotherapeutic treatment of brain, soft tissue, bone, breast and prostate tumours was predicted by changes in MR-DWI. Usually, follow-up studies 2 to 4 weeks after induction of chemotherapy show an increase in the apparent diffusion coefficient (ADC), predicting size changes at the end the treatment cycle. DWI measurements in patients with NSCLC ultra early after starting chemotherapy are missing.

## Patients & methods

23 patients with lung cancer (aged 63.6 ± 7.2 years) underwent serial MRI before and 24h after starting platin-based chemotherapeutical regimens. The MRI protocol contained a DWI sequence with 6 b-values ranging from 0 to 800. Online calculated trace images and the apparent diffusion coefficient (ADC) were used for response evaluation. In 19 patients both clinical information and RECIST evaluations at the end of the second chemotherapy cycle were available.

## Results

18 out of 23 patients showed a decrease in ADC maps 24h after starting treatment.

In three of the 19 patients with available follow-up data, no initial ADC reduction was observed. In all of these patients a progressive disease was observed by the time of completing the second therapy cycle. In 14 patients, initial ADC reduction was associated with tumour size reduction at the end of the chemotherapy cycle.

## Conclusion

Chemotherapy treatment of NSCLC is regularly associated with a decrease in ADC 24h after starting chemotherapy. Initial ADC reduction may predict morphologic tumour response to CHT.

